# Retroperitoneal composite pheochromocytoma-ganglioneuroma : a case report and review of literature

**DOI:** 10.1186/1746-1596-8-63

**Published:** 2013-04-15

**Authors:** Jinchen Hu, Jitao Wu, Li Cai, Lei Jiang, Zhiqiang Lang, Guimei Qu, Houcai Liu, Weidong Yao, Guohua Yu

**Affiliations:** 1 Department of Abdominal Surgery, Affiliated Yantai Yuhuangding Hospital, Medical College of Qingdao University, Yantai, China; 2 Department of Pathology, Affiliated Yantai Yuhuangding Hospital, Medical College of Qingdao University, No.20, Yuhuangding East Road, Yantai 264000, China

**Keywords:** Composite pheochromocytoma/paraganglioma, Retroperitoneal, Ganglioneuroma

## Abstract

**Abstract:**

Composite pheochromocytoma/paraganglioma is a rare tumor with elements of pheochromocytoma/paraganglioma and neurogenic tumor. Most were located in the adrenal glands, and extra-adrenal composite pheochromocytoma is extremely rare. Only 4 cases in the retroperitoneum have been described in the online database PUBMED. Here, we report a case of retroperitoneal extra-adrenal composite pheochromocytoma and review the related literature.

**Virtual slides:**

The virtual slide(s) for this article can be found here: http://www.diagnosticpathology.diagnomx.eu/vs/1700539911908679

## Introduction

Pheochromocytoma arising from outside the adrenal glands is commonly called paraganglioma. But when it occurs below the diaphragm, in the organ of Zuckerkandl or retroperitoneum, it is also called extra-adrenal pheochromocytoma [1]. Extra-adrenal pheochromocytoma is uncommon, and extra-adrenal composite pheochromocytoma is extremely rare. Only 11 extra-adrenal cases have been reported in the literature and 4 of which in the retroperitoneum [2-7]. Most of these rare tumors were compounded with ganglioneuroma. Some of them were functional, resulting in increased level of catecholamines and associated symptoms such as hypertension [6]. However, this case we reported was nonfunctional and the fifth case of composite pheochromocytoma-ganglioneuroma in the retroperitoneum, with the following objectives: 1) to improve understanding of the variable clinical presentation of composite pheochromocytoma-ganglioneuroma, and 2) to identify histomorphological and immunohistochemical features of composite pheochromocytoma-ganglioneuroma.

## Case history

### Case presentation

**Figure 1:**
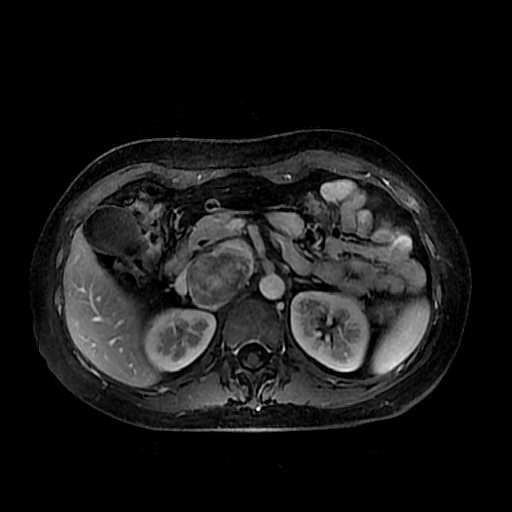
The enhancement scanning of transsected T1-weighted images showed a 6 cm heterogeneous right retroperitoneal mass, with great signal intensity on the edge and weaker in the center, compressing the inferior caval vein and the portal vein.

A 52-year-old Chinese woman was admitted to our hospital for watery diarrhea and febricity for one day, palpitation and debilitation for 6 hours. She had a history of immunologic thrombocytopenic purpura, femoral head necrosis and connective tissue disease. But she had not complained of headache and had no history of hypertension. The sonography of abdomen showed a space-occupying lesion in caput pancreatis. But the magnetic resonance imaging (MRI) (Figure 1) confirmed a 6 cm heterogeneous right retroperitoneal mass, with enhancing rim and central low attenuation on the enhancement scanning of T1-weighted images. The inferior caval vein and portal vein were deformed by the compression of the mass. ST-T abnormalities were seen on the electrocardiogram. The serum carbohydrate antigen 19–9 and carcinoembryonic antigen were normal. And troponin I was 4.4 ug/l, creatine kinase-MB was 15.3 ug/l. During the laparotomy, the retroperitoneal mass lied at the left posterior to hepatoduodenal ligament, right of aorta abdominalis, which compressed forward the inferior caval vein and left renal vein. Then the patient underwent mass resection for biopsy. The findings on pathology examination were consistent with retroperitoneal composite pheochromocytoma-ganglioneuroma. The patient recovered well after tumor resection, without adjuvant chemotherapy or radiotherapy. And Long-term follow-up was suggested.

### Pathologic findings

**Figure 2:**
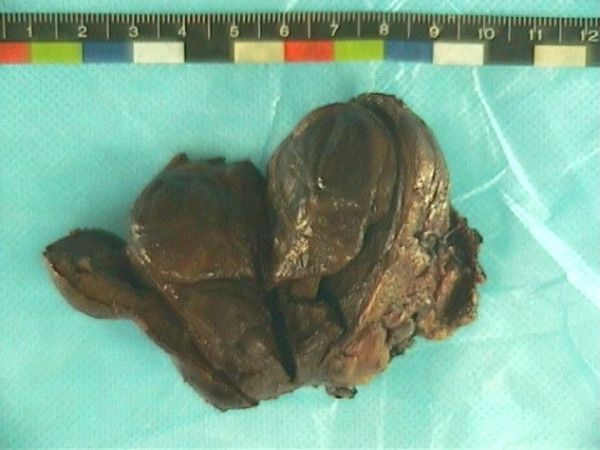
Macroscopic findings: the cut surface of the tumor was soft and dark tan with focal gray-yellow.

**Figure 3:**
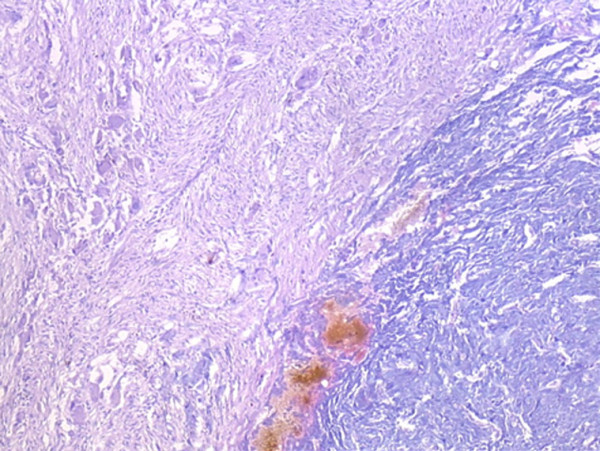
The tumor was composed of two distinct patterns:pheochromocytoma and ganglioneuroma (H&E×40).

**Figure 4:**
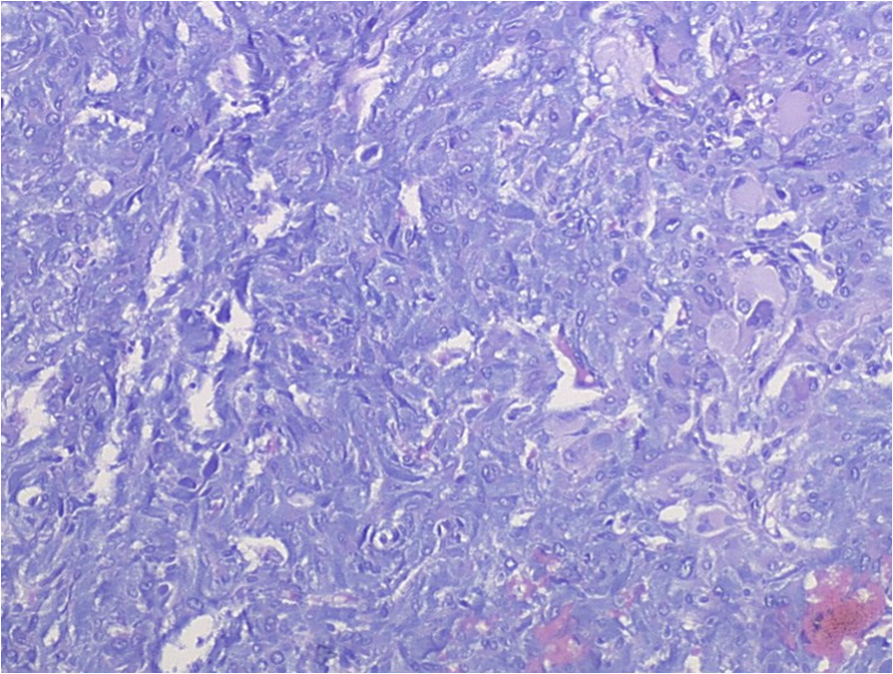
The pheochromocytoma pattern of the composite tumor (H&E×100).

**Figure 5:**
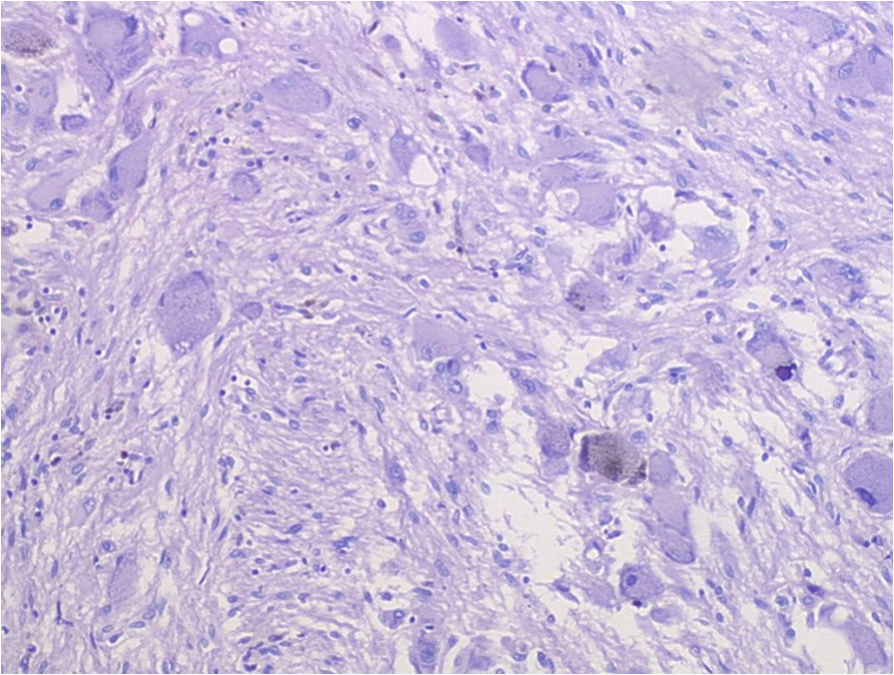
The ganglioneuroma pattern of the composite tumor (H&E×100).

**Figure 6:**
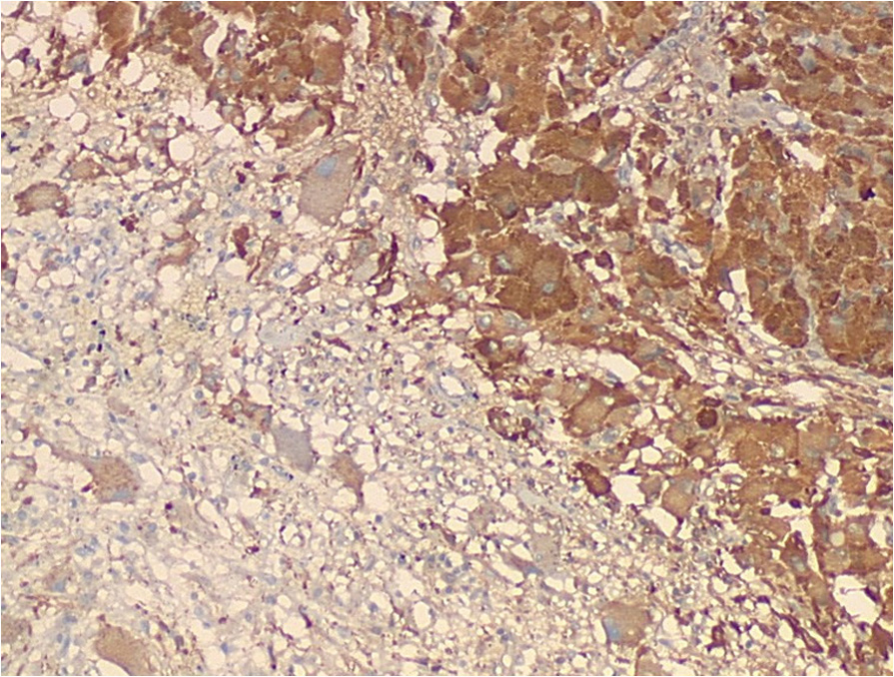
The chromaffin cells were strongly positive for chromogranin A, but the ganglion cells were negative or weakly positive for chromogranin A (Envision×100).

**Figure 7:**
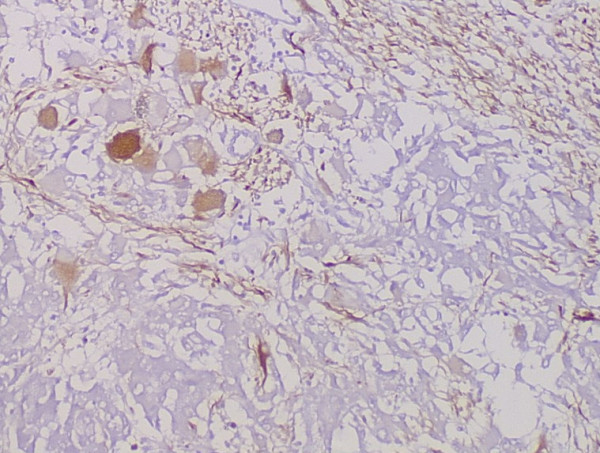
The ganglion cells were strongly positive for neurofilament, but the chromaffin cells were negative for neurofilament (Envision×100).

**Figure 8:**
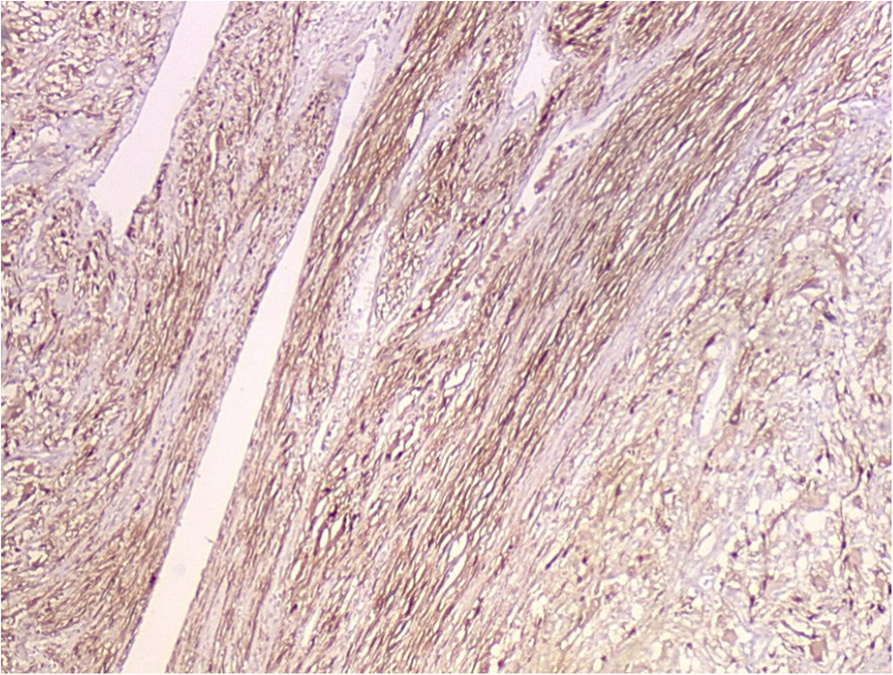
The sustentacular cells of the pheochromocytoma component and the schwannian cells of ganglioneuroma component showed characteristic staining of S100 (Envision×40).

Grossly, the external surface of soft solid tumor was smooth and well-encapsulated. The tumor measured 6 cm × 5 cm × 4 cm. The cut surface was soft and dark tan with focal gray-yellow (Figure 2). Histologically, the tumor was composed of two distinct patterns (Figures 3, 4, 5), stained with hematoxylin-eosin (H&E). One pattern was consistent with pheochromocytoma. It was composed of nests of large, polygonal and pleomorphic cells with granular and basophilic to amphophilic cytoplasm. And the nuclei were round to oval with prominent nucleoli. The nests were separated by spindle-shaped, elongated cells (Schwann cells) (Figure 4). The other pattern consisted of ganglion cells embedded in the Schwann cell which was consistent with ganglioneuroma (Figure 5). The two components were separate but irregularly merged from one into the other. The necrosis or mitotic figures were not found. But in some focal areas, some small round primitive neuroblastic-like cells were present. Immunohistochemistrical staining was performed by the streptavidin-biotin-peroxidase method and diaminobenzidine as chromogen. Primary antibodies used in this study were prediluted and bought from Maixin Biocorpration, China. Appropriate positive and negative controls were performed. Both the pheochromocytoma component and ganglioneuroma component of the tumor were stained positively for neuron-specific enolase (NSE), synaptophysin (Syn) and Vimintin(Vim). The chromaffin cells were strongly positive for chromogranin A and negative for neurofilament (Figures 6, 7). However, the ganglion cells were negative or weakly positive for chromogranin A, but positive for neurofilament (Figures 6, 7). The sustentacular cells of the pheochromocytoma component and the schwannian cells of ganglioneuroma component showed characteristic staining of S100 (Figure 8).

## Discussion

Composite pheochromocytoma/paraganglioma is a rare tumor that typically combines features of pheochromocytoma or paraganglioma with those of ganglioneuroma, ganglioneuroblastoma, neuroblastoma, peripheral nerve sheath tumour or neuroendocrine carcinoma [8]. Fewer than 70 cases have been reported in the medical literature. Most were located in the adrenal glands, and extra-adrenal composite pheochromocytoma/paraganglioma was reported occasionally [9]. To the best of our knowledge, only 11 extra-adrenal cases have been reported, located in the urinary bladder (6 cases) [2], the retroperitoneum (4 cases) [3,4,6,7], and the cauda equine (1 case) [5], respectively.

About 71% of composite pheochromocytoma/paraganglioma coexisted with ganglioneuroma (Pheo-GN). The patients ranged from 5 to 82 years old, with the majority in the age range of 40 to 60 years, and these tumors occurred with approximately equal frequency in male and female subjects [9]. The 4 patients with composite pheochromocytoma/paraganglioma in the retroperitoneum were older than 40 years of age (range 41-63 years). Males and females are equally affected. All of which coexisted with ganglioneuroma. 2 cases have been reported in Japan, with the rest in China and USA (Table 1).

**Table 1:** Clinical and pathologic features in four cases of retroperitoneal composite pheochromocytoma

**Age/Sex**	**Clinical Features**	**Syndrome**	**Tumor Size(cm)**	**Histopathology**	**Outcome**	**Reference(country)**
48/M	NA	NA	NA	PH-GN	NA	Yoshimi et al.(5),1992(Japan)
41/F	Hypertension,palpitation,headache, retroperitoneal mass	None	4.4 × 4 × 2	PH-GN	Asymptomatic and tumorfree for one year	Tohme et al.(4),2006(USA)
63/F	retroperitoneal mass	None	6.5 × 5 × 3	PA-GN	Tumorfree for 18 months after surgery	Hirasaki et al.(6),2009(Japan)
50/M	retroperitoneal mass	None	4.5 × 4 × 2.5	PA-GN	Tumorfree and in good condition for over 36 months	Jing et al.(7),2010(China)

NA, not available; PH, pheochromocytoma; GN, ganglioneuroma; PA, paraganglioma.

Like ordinary pheochromocytoma/paraganglioma, most cases of composite pheochromocytoma/paraganglioma were functional, with increased level of catecholamines or corticotropin-releasing hormone (CRH) [10] or their metabolites or whatever, and over 62% of the patients were also hypertensive [3]. Embryologically, both the chromaffin cells of the pheochromocytoma and the ganglion cells of the ganglioneuroma are derived from neural crest cells and migrate to somatic areas [9]. These cells have the potential to produce many peptide hormones and biogenic amines [11]. It is apparent that the composite tumor of pheochromocytoma and ganglioneuroma may display symptoms referable to hormonal hypersecretion by either portion of the tumor, such as headache, palpitation, and excessive perspiration. And Mahajan et al. [11] described watery diarrhea attributable to increased secretion of vasoactive intestinal peptide (VIP) in some cases. Both the phaeochromocytoma and non phaeochromocytoma components were capable of secreting VIP. This further supported its common derivation. In the 4 cases of retroperitoneal composite pheochromocytoma/paraganglioma, only 1 case was found functional evidence, such as hypertension, headache or palpitation, preoperatively [6]. Another case was reported that the ganglion cells in ganglioneuroma were positive for VIP in immunohistochemical examination [7]. In our case, the patient had no hypertension or any classic symptoms of pheochromocytoma. She only complained of palpitation and debilitation. However, watery diarrhea may be caused by the composite tumor through secreting VIP.

The composite pheochromocytomas/paragangliomas were often associated with familial neoplasm syndromes such as neurofibromatosis type 1 (NF1) [12] or multiple endocrine neoplasia type 2A (MEN 2A) [13]. However, as in our case, association of composite tumor with NF1 or MEN 2A had not been reported in the 4 cases of retroperitoneal composite pheochromocytoma/paraganglioma.

The size of composite pheochromocytoma/paraganglioma ranged from 1 to 35 cm, with the average being 4 to 6 cm. And the size of which in the retroperitoneum ranged from 2.0 to 6.5 cm in diameter. Grossly, composite pheochromocytomas/paragangliomas were usually similar to typical pheochromocytomas. So the composite phenotype was generally detected during histological examination. Gross examination of the tumors usually revealed patchy, pale areas corresponding to ganglioneuroma or necrotic and hemorrhagic areas of ganglioneuroblastoma sometimes [14]. The different cell populations were admixed or arranged in a patchwork as a collision tumor. The presence of stromal features hinted the mixed phenotype. In our case, the tumors were traversed by bundles of spindle cells that shown containing Schwann cells and axons immunohistochemically, and there were also patchy areas with prominent sustentacular cells.

Immunohistochemically, the individual components of these tumors resemble their normal counterparts or pure tumors of the same type. Both Chromogranin A and synaptophysin were strongly and diffusely positive in the pheochromocytoma/paraganglioma cells [15]. In contrast, staining was weak or focal in neuroblasts and mature neurons. This difference was helpful in identifying foci of neuroblastoma in composite tumors and for distinguishing neuroblasts from mature pheochromocytoma cells of the same size. Immunoreactive catecholamine-synthesizing enzymes such as tyrosine hydroxylase (TH) and phenylethanolamine N-methyltransferase (PNMT) were helpful to identify catecholamine-producing cells and infer the specific catecholamines they produced [9]. In addition, staining for S-100 protein identifed Schwann cells and sustentacular cells. While staining for neurofilament proteins aided in identification of axon-like processes. Immunoreactive VIP was found predominantly in neuronal components of the composite tumors with watery diarrhea-hypokalemia-achlorhydria syndrome. However, VIP was also positive in phaeochromocytoma cells occasionally [11,16].

No genetic abnormalities were found to distinguish composite tumors from pure pheochromocytomas or paragangliomas. Comstock et al. [17] reported that neither composite pheochromocytomas nor classic pheochromocytomas demonstrated N-myc amplification. But 5 (50%) of 10 neuroblastomas showed significant N-myc amplification. These findings suggested that N-myc amplification in the composite pheochromocytoma with neuroblastic elements may imply worse outcome.

It was difficult to predict the biologic behavior of composite tumors. The prognosis associated with composite phaeochromocytoma-ganglioneuroma was variable. Metastatic lesions from these tumors were almost derived from the neural component; however, pheochromocytoma metastasizing as a single entity or in conjunction with the malignant neural component was found in some uncommon cases [16]. Only 1 case of phaeochromocytoma-ganglioneuroma had been reported with liver metastatic lesions at the time of autopsy [16]. In the 4 cases of retroperitoneal composite phaeochromocytoma-ganglioneuroma, the invasion of the inferior vena cava and of the right renal artery in one case was concerned about the malignant potential. But the patient was asymptomatic and tumorfree after one year of follow up [6]. And the malignant features such as infiltration or metastasis were not found in the other cases. In our case, the presentation of some small round primitive neuroblastic-like cells in some focally areas maybe imply malignant potentiality prognosis. However, the patient was tumorfree for 24 months after surgery.

Composite pheochromocytoma/paraganglioma-ganglioneuroma were treated in principle by surgical resection, as most of which were benign [2,9]. And adequate clinical follow-up was advised for the potentially malignant neoplasms.

In conclusion, composite pheochromocytomas/paragangliomas were rare tumors most located in adrenal glands. And extra-adrenal composite pheochromocytomas/paragangliomas were extremely rare. The clinical features were similar to ordinary pheochromocytoma, but little was known about the biologic behavior, outcome or molecular genetic profile. Herein, we described a retroperitoneal composite pheochromocytoma coexisted with ganglioneuroma and reviewed the related literature. Although composite pheochromocytoma -ganglioneuroma in the retroperitoneum was not common, the possibility of a neuroendocrine tumor should be considered in the differential diagnosis of a retroperitoneal mass, especially in patient with hypertension or watery diarrhea.

## Consent

Written informed consent was obtained from the patient for publication of this report and any accompanying images.

## Competing interests

The authors declare that they have no competing interests.

## Authors’ contributions

JH carried out the operation and drafting the manuscript about clinical data. JW carried out the follow-up and participated in drafting the manuscript about the pathological data. LC carried out searching the literature in the online database PUBMED and participated in drafting the discussion of manuscript. LJ carried out immunohistochemistrically sectioning and staining. ZL carried out fixing with 10% formalin, embedding in paraffin, sectioning and staining with HE. GQ participated in immunohistochemistrical observation and analysis. HL participated in gross pathological and histological observation and analysis. WY participated in gross pathological, histological and immunohistochemistrical observation and analysis. GY carried out collecting the pathological image and revising it critically for important content. All authors read and approved the final manuscript.
